# Chemistry and Its Application to Dentistry

**Published:** 1853-01

**Authors:** A. Snowden Piggot


### ARTICLE IX.

*Chemistry and its Application to Dentistry.* By A. SNOWDEN PIGGOT, M. D.

IN the rapid advance of the sciences which characterizes this century, chemistry has held a very prominent place. Indeed, a history of the progress of human intellect, of arts, of sciences, during the last fifty years, would be, to a great extent, a history of chemistry. It has so interwoven itself with almost every thing that we either think or do, that it is much harder to determine what department of practical or speculative exertion is independent of this science than what absolutely needs it. The astronomer, when looking deep into infinity with almost angelic ken, cannot emancipate himself from the influences of chemistry. The photographic apparatus with which he makes the heavens themselves record his observations for him, as well as the glasses by whose aid he penetrates the deep abyss of worlds, are alike furnished him by this assiduous handmaid of human improvement. The geologist, whose researches are to time what the astronomer's are to space, feels the same need of this universal alphabet of science. His interpretation of the stony hieroglyphics, in which the great cycles of creation have chronicled their history as they passed, would stop, for want of a key, just where the interest was becoming most intense, did not chemistry hold out her lamp and show the anxious inquirer the secret processes of the world's great laboratory.

If we turn from science to art, from the speculations of the student to the every day duties of life, we shall still meet this omnipresent science at every turn. Our houses are warmed and lighted, our clothes are dyed, our linen is bleached, our bodies are cleansed, our food is prepared, our condiments are made, our necessaries, our comforts, our ornaments, our luxuries are obtained by processes which the chemist has either originated, cheapened or greatly improved. Even in those arts which seem, at first sight, farthest removed from the supervision of physical science, chemistry claims the right of intervention. It quickens the operations of commerce and strengthens the arm of justice. When the law reaches its avenging arm over half a continent and seizes the guilty wretch, in the midst of his fancied security, a thousand miles from the scene of his crime, how few recognize the fact, that this increased facility of detecting villainy and the consequent additional protection of the innocent from the craft or violence of guilt, are the result of a practical application of the chemist's researches and discoveries.

In no department of science or art has this study of the affinities of bodies been productive of more brilliant results than in physiology, pathology and therapeutics. The experimentalist who has been pursuing the protean manifestations of life, till he has almost surprised the plastic power, in the midst of her secret operations, has been guided in every step of his progress by this faithful and sagacious assistant. The researches of Berzelius, Prout, Liebig, Dumas, Mulder, and a host of others have shed a new light on physiology. The Protein discussion, let the question be decided ultimately as it may, has done very much towards the establishment on a firm basis of the theory of the chemical development, degeneration and final disintegration of the tissues. The collateral questions it has originated, the incidental discoveries to which it has led, are of themselves of no little importance to the science of life. It is to the stimulus which this great controversy gave to research in this particular department, that we owe the discovery of that interesting compound, Leucine, which seems to furnish the basis of future inquiries into the chemical origin of the gelatinous tissues. But it would be impossible in any space, short of that of a treatise on animal chemistry, to enumerate the benefits which that science has conferred on physiology. It has, indeed, remodeled the whole thing. Without modern chemistry, modern physiology could not exist.

The sect of the pure vitalists may, indeed, be considered as extinct, so far as their influence over the minds of the earnest students of healthy structure and function is concerned. Here and there, indeed, we find a sturdy champion of the old faith, zealously battling against what he considers the unwarrantable innovations of the chemist, but his opposition is usually grounded on a mistake or a misconception of the views he contests. It is a man of straw of his own manufacture over which he gains his victories. He asserts that the chemical physiologist wishes to resolve all vital actions into the mere play of chemical affinities, entirely setting aside and putting out of view the vital force, and he strenuously combats this theory. In reality, however, his opponent is as fully convinced as any one can be of the existence of this vital force, and it forms as important an element in his calculations as affinity itself. The real difference between them is, that the modern physiologist, in the true spirit of scientific inquiry, desires to extend the domain of second causes to its utmost possible limit, while, the vitalist, with the timidity of superstition, (for there is superstition in science as well as in religion) clings to old dogmas, fears to venture beyond the shadow of some venerable name, and regards the whole broad field as holy ground, sacred to the memory of the vital principle. Thus the latter is cut off from all improvement, while the former, emancipated from the dread of this unknown god, vigorously pushes his researches in all quarters where any mine of truth may be suspected to be hid. He, too, reaches a point where he is compelled to own the existence of a force totally unknown to dead matter, but even then he fully recognizes the applicability to science as well as to art of the old Horatian maxim:

"Nec Deus intersit, nisi dignus vindice *nodus*."

In pathology, too, chemistry has rendered great and acknowledged service to medical science. The intricate metamorphoses of tissue, preparatory to excretion by the kidneys, the influence of diet upon these, and the changes which a slight aberration from normal action may effect in the ever-varying excreted fluid, have been disclosed to the physician by chemistry. As formerly, there was scarcely any chain of morbid actions so obscure as that which resulted in urinary deposits or other disturbances, so now there is hardly any more thoroughly understood. If time and space were allowed, many other equally striking examples of the improvement which pathology owes to chemistry might be adduced, but our limits compel us to confine ourselves to what we have already cited.

The contributions of chemistry to therapeutics may be classified under two heads: first, the improvement in elegance, concentration, purity and cheapness of the articles of the materia medica; and, secondly, the insight into healthy and diseased function and into the nature of therapeutic power, which leads of course to a more accurate and scientific *methodus medendi.* The introduction of the alkaloids need only be alluded to. Every one knows the great benefit they have been to the practitioner of medicine. Curative potencies, that were formerly available only in powders by the table-spoonful or potions by the pint, are now administered in drops or pillicules to the size of which a homeopathist could not object. The easy detection of adulterations, too, is not the least benefit which the progress of chemistry has conferred upon the purchaser of medicines. In the second department, viz. the improvement of the *methodus medendi,* a candid writer must acknowledge that chemistry should be spoken of rather as doing than as having done service. And yet it has left its mark here too. The management of urinary disease has certainly very much improved since the beginning of this century, and the advance is due entirely to the influence of chemical facts and theories. A new light has been thrown also on the *modus operandi* of some cholagogues. In other classes of remedial agents we can discern signs of the coming of improvement. But, in the introduction of new remedies, chemistry, certainly, has won great triumphs. This is the age of chloroform and the ethers. Their introduction marks a new era, as well in surgery as in medicine, and chemistry has brought them in.

It is somewhat singular that, while general medicine has received so many acknowledged benefits from this beautiful science, the special department of dentistry has paid so little attention to the great stir of mind around it. This neglect of chemistry on the part of the dentist is still more surprising, when we consider that he has to deal with that solid portion of the animal frame, which, of all others, is most liable to easily discernible chemical changes. The teeth are further removed from the vital influences of the system than any other portion of the human body except the hair. A tooth grows once for all, and let its integrity be once seriously impaired, it must be considered and treated as a foreign body. It possesses no power of reparation, for the slight effusion of callus occasionally observed on the surface of denuded dentine cannot be rated under the head of true recuperative processes. Hence it is peculiarly liable to extraneous injuries from which it can neither protect itself nor recover. The chemical causes which may interfere with its health, (and they constitute by far the larger division of dental etiology,) cannot be understood without a thorough knowledge of the composition of the teeth, of the soft parts which surround them, of the secretions which bathe them, and of the food which they masticate. The elementary constitution of the teeth themselves must of course form the basis of every inquiry of the kind, because it is the action of foreign agents upon their structure which the dentist is chiefly anxious to ascertain.

This point having been determined, it next becomes necessary to comprehend the composition of the fluids of the mouth and their normal relations to the teeth, for we cannot understand disease, which is nothing more than aberration from healthy function or structure, without a previous knowledge of health. The changes which the saliva undergoes from the varying admixture of buccal, palatal and labial mucus, changes induced by the diminution or the increase of one or other of these secretions, should be examined, for they are by no means unimportant in the study of dental pathology. It is necessary also to know the modifications which the various diseases of the soft parts are capable of producing in these liquids. The different salivary diseases exert their influence over the teeth through the medium of the chemical changes they effect in the saliva, and as these are numerous and varied, the whole domain of salivary pathology must be explored by him who would gain a competent knowledge of these little organs, so essential to our comfort and beauty. The food must be studied not merely in relation to its atomic constitution, but also in reference to the compounds which may be generated in it by fermentation and putrefaction, for that part of it which is retained in the crevices of the teeth is subjected to those very conditions of heat, moisture and atmospheric agency which are most efficient in inducing these changes, and some of the acids generated by these processes are capable of acting as solvents upon both the enamel and the dentine.

But this would be a very imperfect insight into the physiology of the teeth. There remains still a most important investigation. The teeth and mouth are the vestibule of the alimentary canal. In and by them are performed the preliminary processes of digestion, and these have a direct and intimate relation with the actions of the stomach. Imperfections in the teeth interfere with mastication and so throw upon the stomach a burden which that organ is not always able to sustain. Disorder of the salivary secretion materially affects the subsequent processes of digestion. So, too, the stomach may react upon the mouth. Disease in that viscus is almost sure to induce disease in the latter cavity. Whether this be structural or functional, it can hardly fail to produce some alteration in the chemical constitution of the fluids, which, in turn, may act upon the teeth. A thorough study of the chemistry of the mouth is not, therefore, possible without a general survey of the whole great function to which the parts in the cavity of the mouth are subservient.

But the duty of the dentist does not end with a theoretical knowledge of the physiological functions and relations, and the pathological alterations of the teeth. It is his business to repair those injured parts which unaided nature is incapable of restoring. He must, therefore, thoroughly understand the materials with which these repairs are to be made. He must know the action of the healthy and diseased fluids upon the various metals with which he proposes to fill the cavities left in the teeth by caries, as well as the chemical effect which the substances introduced to destroy the nerve may have upon the denuded dentine. Without some such knowledge as this, it would not be difficult for him to do much harm in trying to do a little good. It is quite possible to introduce a filling into a cavity which will act as a poison upon the entire mouth. It is quite possible to inflict irreparable injury upon a tooth while attempting to destroy a nerve. In fact the ability of ignorance to injure is unlimited, while the power of knowledge to repair is confined within very narrow bounds.

The chemist's aid is also needed by the dentist in his plate work. The theory of alloys, the amount of silver or base metal which it is advisable to introduce into the gold with which plate-work is to be done, must be known by the skillful artificer. But the mechanical dentist does not use pure gold in his operations. His gold, for these purposes, as well as his silver, is generally obtained from coin. This is an alloy perpetually varying with the price of labor, the relative value of the metals composing it, the caprice of assayers, the skill of mint-masters, and the honesty of governments. The coinage of a nation in any given year, however, is generally uniform, so that the chemist's aid is needed again by the dentist in this particular, to inform him of the exact composition of the coinage of different years and nations. Again, these metals are of variable volatility, escaping into the atmosphere at different degrees of heat. To get an alloy, therefore, which shall be uniform, allowance must be made for the volatilization of the components of the metallic bath, a task of some difficulty and requiring no little familiarity with metallurgic operations.

If the manufacture of block and other artificial teeth is to be conducted with success, the aid of the chemist is again required. The minerals used for them, are, themselves, compounds, containing various proportions of silica and the earths. A judicious combination of them is impossible without an acquaintance with their constituents, and their mutual reactions at a high temperature, when the usual affirmatives of bodies are greatly modified. The principles of fluxing must also be known in order to secure an even enamel, the polish of which shall be neither too bright nor too dull for a good imitation of nature.

These teeth, too, must be colored to resemble the natural organs. The common white of untinted porcelain will not answer, for it is altogether unlike the white of a tooth. The latter has different tints of pearly gray, blue, green and yellow, which must be imitated by the metallic oxyds. The study of the colors produced by these and of the delicate modifications of which, under judicious management, they are susceptible, is almost a distinct department of chemical inquiry. Any one who has examined a well-made plate of Sevres or Berlin china knows very well the exquisite delicacy and gradation of tint, which the chemist has rendered possible to the artist. The dentist must apply the same principles, which have led to such beautiful results in the porcelain manufacture, to the production of the faint tints, and the blending of colors which shall correctly counterfeit the hues of healthy teeth and gums. The proper fluxing of the different oxyds must also be carefully studied as it may influence the tint.

This is a hasty and imperfect outline of the points at which chemistry and dentistry touch one another. Its filling up will be a subject for future consideration. As it is, it is offered to the dental student as a hint to guide him in the prosecution of the chemical studies. One of our dental schools has already recognized the necessity of this addition to its curriculum, and the beneficial effects of a well digested and carefully conducted course of instruction in this department cannot fail to be felt by the student. We doubt not that the change will meet the approbation of all who desire the improvement of the present system of dental education, for every practical and thinking man must have felt the necessity of some such chemical knowledge as we have pointed out in our brief remarks.

